# Automated image segmentation-assisted flattening of atomic force microscopy images

**DOI:** 10.3762/bjnano.9.91

**Published:** 2018-03-26

**Authors:** Yuliang Wang, Tongda Lu, Xiaolai Li, Huimin Wang

**Affiliations:** 1 School of Mechanical Engineering and Automation, Beihang University, Beijing 100191, P.R. China; 2 Beijing Advanced Innovation Center for Biomedical Engineering, Beihang University, Beijing, 100083, P.R. China; 3 Department of Materials Science and Engineering, Ohio State University, 2041 College Rd., Columbus, OH 43210, USA

**Keywords:** atomic force microscopy, contour expansion, image flattening, polynomial fitting, sliding window

## Abstract

Atomic force microscopy (AFM) images normally exhibit various artifacts. As a result, image flattening is required prior to image analysis. To obtain optimized flattening results, foreground features are generally manually excluded using rectangular masks in image flattening, which is time consuming and inaccurate. In this study, a two-step scheme was proposed to achieve optimized image flattening in an automated manner. In the first step, the convex and concave features in the foreground were automatically segmented with accurate boundary detection. The extracted foreground features were taken as exclusion masks. In the second step, data points in the background were fitted as polynomial curves/surfaces, which were then subtracted from raw images to get the flattened images. Moreover, sliding-window-based polynomial fitting was proposed to process images with complex background trends. The working principle of the two-step image flattening scheme were presented, followed by the investigation of the influence of a sliding-window size and polynomial fitting direction on the flattened images. Additionally, the role of image flattening on the morphological characterization and segmentation of AFM images were verified with the proposed method.

## Introduction

Since its invention, the atomic force microscopy (AFM) has become an important device in the fields of nanoscale imaging [1–6], nanoscale manipulation [7–8], and material property characterization [9–13] because of its ultra-sensitivity in force and displacement measurement. Among the different applications, imaging is the most basic one. Various samples, such as molecules [14–15], live cells [1], and interfacial nanobubbles (NBs) [16–17] can be imaged with AFM, in environments ranging from ambient to liquid, or even vacuum.

**Figure 1:**
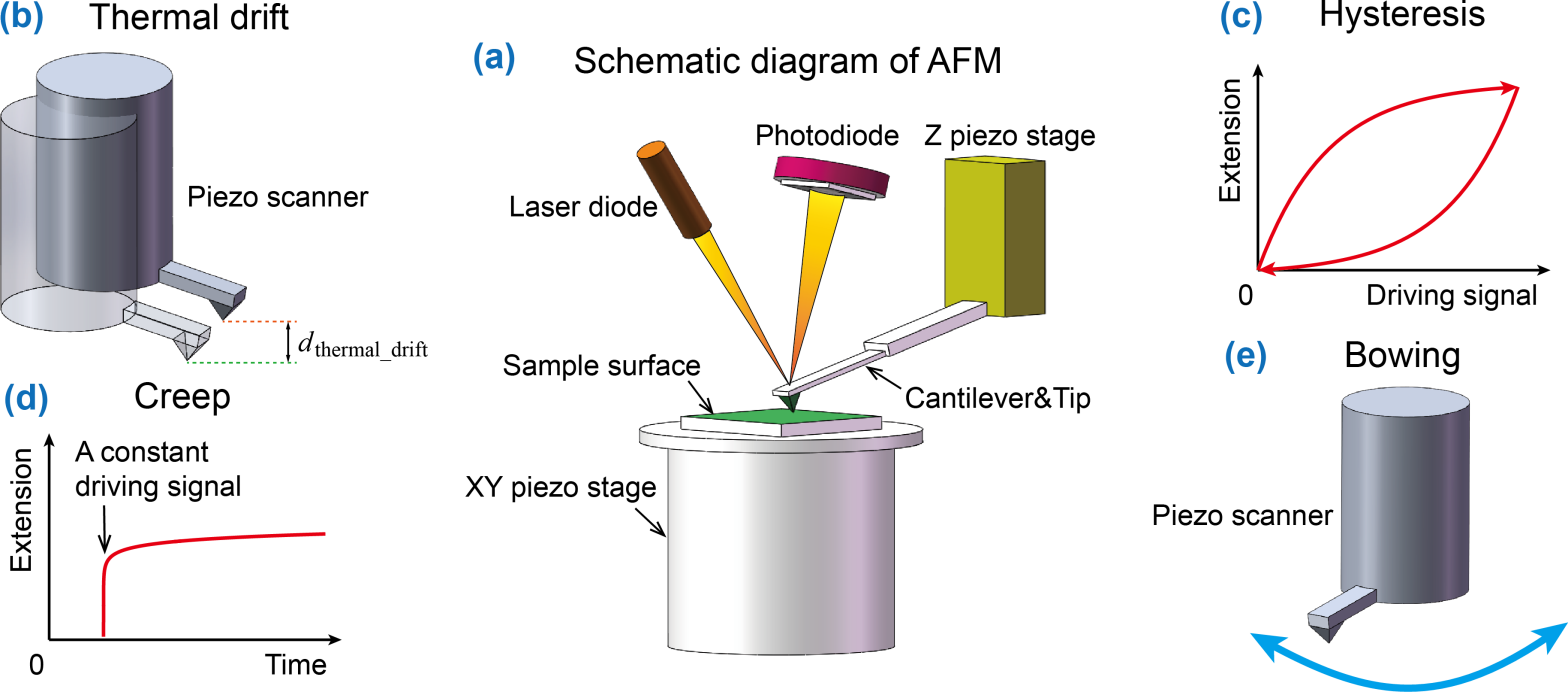
Schematic diagram of an AFM system and mechanism behind the distortion and artifacts present in AFM images. (a) Schematic diagram of an AFM system, consisting of an *x*–*y* and a *z*-scanner. (b–e) Mechanism of distortion and artifacts caused by thermal drift, hysteresis, creep, and nonlinearity of a scanner.

Sample images obtained from AFMs can be compromised by distortion and artifacts, which are mainly caused by mechanical drift in AFM systems [18–21]. As illustrated in Figure 1a, an AFM system is generally composed of two piezo-driven stages, an *x*–*y* sample stage and a *z*-scanner. The *x*–*y* sample stage is used to implement precise lateral motion for point-by-point scanning while the *z*-scanner adjusts the vertical position of the AFM cantilever substrate to maintain constant interaction between the cantilever tip and sample surface. Together, the two stages provide a three-dimensional (3D) topographical reconstruction of the sample surface. However, the obtained images are unavoidably influenced by the mechanical drift between the two stages (Figure 1b). Using the visual sensing approach, Wang et al. demonstrated that the vertical drift between the two stages can be up to 600 nm within a 30 min time period [21]. Therefore, mechanical drift becomes a major source of artifacts exhibited in AFM images. Additionally, some other factors, such as hysteresis (Figure 1c), creep (Figure 1d), and nonlinearity (Figure 1e) of *x*–*y* and *z*-scanners and vibration from the environment can also cause distortion and artifacts in AFM images [20,22–24].

AFM images generally display a tilting, bowing, or other types of low frequency image artifacts [19,21,25–27]. Among the different types of artifacts, those showing specific frequencies can be eliminated through fast Fourier transform (FFT) methods [28]. However, for some other artifacts, such as tilting and bowing, flattening is required prior to analysis.

In AFM image flattening, individual scan lines are fitted as polynomial curves with the least-square method [29]. The obtained polynomial curves are then subtracted from AFM scan lines to get flattened images. The direct polynomial fitting can cause stripe-type artifacts, associated with the concave or convex features in AFM images. To obtain optimized results, flattening with excluded mask areas can be applied [3,30]. In this method, the concave or convex features are first masked and excluded in the raw images. The polynomial fitting is only applied to the unmasked portion of scan lines or images. As a result, stripe-type artifacts that appear in the direct polynomial fitting based flattening can be avoided. The method is referred to as mask exclusion flattening (MEF) in this study.

MEF consists of two major steps, extraction of exclusion masks and polynomial fitting of the image background. Currently the extraction of exclusion masks in MEF is implemented manually. Rectangles and ellipses are taken as masks to select features of interest. However, they are not in accordance with the features to be excluded, regarding size and shape. Moreover, as is often the case, hundreds of features maybe required to be excluded in one image, which makes the manual operation tedious and time consuming. A robust and automated algorithm for feature detection in MEF is highly awaited.

Essentially, the extraction of the exclusion masks is a process of image segmentation, whereby methods including thresholding [31–32], circle Hough transform [33], and clustering [30] can be applied. Recently, Wang et al. proposed a contour expansion method for feature extraction in AFM height images [3,34]. The method achieves an accurate localization and optimized boundary detection for foreground features in AFM height images.

Regarding the polynomial fitting of the scan lines, when the artifacts and distortion in AFM images are more complex than tilting or bowing, the direct polynomial fitting will not guarantee desired background elimination [35]. This is because the individual scan lines can no longer be represented by polynomial curves.

To solve the problems mentioned above, a two-step scheme was proposed for optimized flattening of AFM images in this study. In this method, the contour expansion method [3] was first applied to achieve automated extraction of exclusion mask areas for features in the foreground. Then, polynomial curve and surface fitting were applied to the portion of the image in the background. For images with complex artifacts, sliding-window-based polynomial fitting was applied to obtain optimized flattening results.

The sections of this paper are organized as follows. The Experimental section introduces the sample preparation and imaging of surface nanobubbles using an AFM. In the section Methods, the exclusion mask extraction and polynomial fitting will be presented individually. In the section Results and Discussion, the comparison of different flattening methods will first be conducted. The sliding-window-based MEF is then introduced, followed by an investigation of the influence of sliding-window size and fitting direction on the flattened images. Finally, the role of image flattening on morphological characterization and image segmentation of AFM images is demonstrated.

## Experimental

There are normally hundreds of NBs in one AFM image, which make it challenging to conduct image segmentation manually. In this study, NB images were taken as examples to validate the proposed scheme of image flattening. To obtain NBs, a sample was prepared by spin coating a thin film of polystyrene (PS) on a silicon (100) substrate at a speed of 500 rpm. Prior to spin coating, the silicon substrate was sequentially cleaned in a sonic bath of piranha solution, acetone and water for 30 minutes. The PS solution was obtained by dissolving PS particles (molecular weight 350,000, Sigma-Aldrich) in toluene (Mallinckrodt Chemical) to a concentration of 0.2%. During the experiment, the PS film was immersed in deionized (DI) water. The NBs spontaneously nucleated on the PS surface. Besides NBs, a PS film covered by nanopits was taken as an example of convex features for image flattening. Additionally, a standard calibration grating (10 µm-pitch grid, Bruker) with a nominal depth of 180 nm was also applied to demonstrate the role of image flattening on morphological characterization of AFM images.

All AFM images used in this study were obtained on a commercial AFM (Resolve, Bruker) in tapping mode with 96% setpoint value. A silicon cantilever (NSC36/ALBS, MikroMasch) with quoted stiffness of 0.6 N/m and tip radius of 8 nm was used for scanning. The scanning frequency and scanning angle were 2 Hz and 0°, respectively.

### Methods

**Figure 2:**
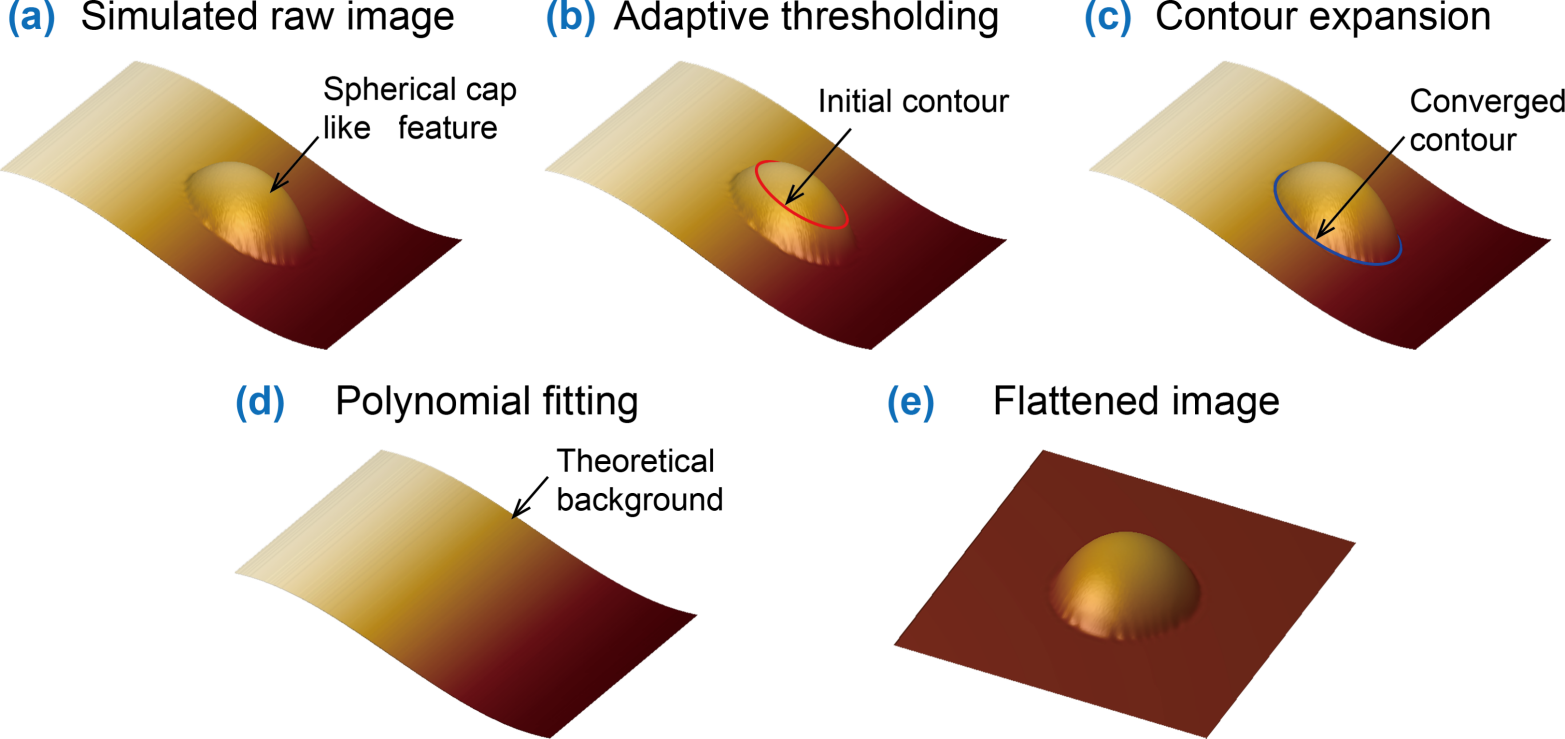
Illustration of the proposed method for AFM image flattening. (a) Sketch of a simulated AFM height image with a tilting background. (b) Preliminary detection of a feature in the foreground using the adaptive thresholding method. (c) The optimized boundary detection through contour expansion operation to determine the foreground mask area. (d) The obtained surface by fitting the background as a polynomial surface after exclusion of the foreground mask area. (e) The flattened image obtained by subtracting the polynomial surface from the raw image.

The step-by-step procedure of the proposed two-step AFM image flattening is illustrated in Figure 2. In the illustration, a simulated AFM image with a spherical-cap-like object and tilting background is constructed (Figure 2a). The adaptive thresholding [36] is first applied to get a preliminary contour (red contour in Figure 2b) of the spherical object. The contour expansion operation [3,37] is then conducted to achieve the optimized boundary detection (blue contour in Figure 2c). After that, the area enclosed by the detected contour is taken as the exclusion mask. The remaining area is taken as the background. The polynomial fitting is applied to the background to get a theoretical surface (Figure 2d), which represents the trend of the background. The obtained theoretical surface is then subtracted from the raw image to obtain the flattened image, as shown in Figure 2e.

### Extraction of exclusion areas in foreground

**Figure 3:**
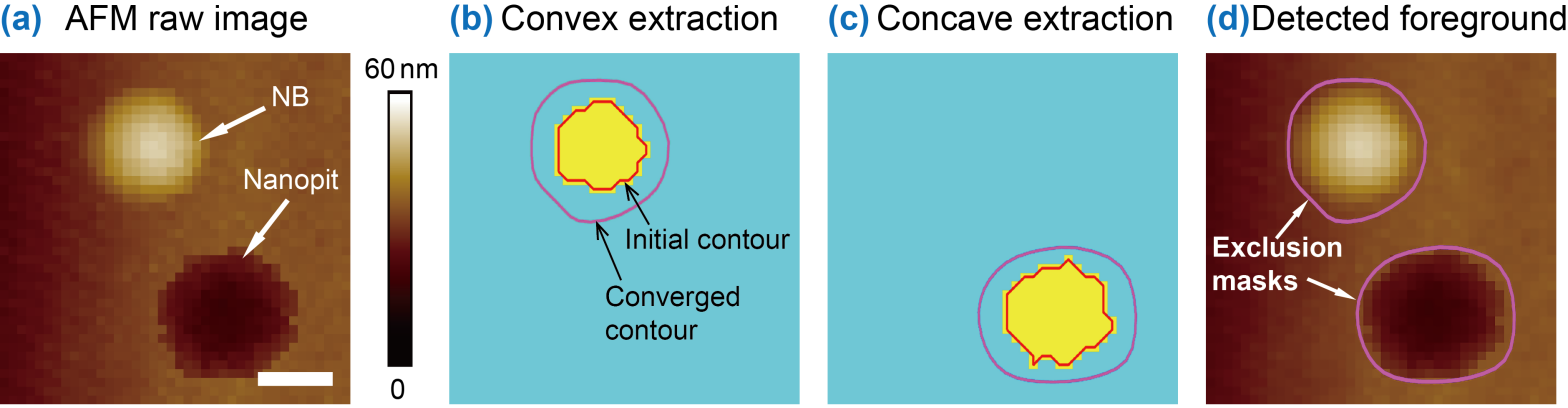
Demonstration of exclusion area extraction through image segmentation. (a) A raw AFM image with a convex (NB) and concave (nanopit) feature. (b) and (c) show mask images of the NB and nanopit, respectively. The red curves are initial contours of the features obtained by adaptive thresholding, while the purple curves indicate the optimized boundaries obtained by contour expansion. (d) AFM image with the detected boundaries of the two features. It is clear that contour expansion can achieve good estimation of the features in the foreground. The scale bar is 100 nm.

In the proposed image flattening operation, the automated extraction of features in the foreground is a primary operation. Both convex and concave features were extracted from AFM images as exclusion masks. Figure 3a shows a raw AFM height image. In the image, there is one NB (convex feature) and one nanopit (concave feature). The adaptive thresholding was first applied to the image to get a preliminary segmentation of the NB (yellow mask area in Figure 3b). The boundary of the mask area was then extracted as the initial contour (red contour). Driven by the gradient field of the raw image, the contour gradually evolves until it converges to the actual boundary of the NB [3,37–38], as indicated by the purple curve in Figure 3b.

To detect the concave feature, the complement of the raw image was first obtained. In the complement image, the height value of the concave feature was reversed. The contour expansion operation was applied to the complement image and then concave feature was segmented (Figure 3c). The detected boundaries for the NB and nanopit are shown in Figure 3d. The enclosed areas within the detected boundaries are taken as exclusion masks for MEF.

Here readers should note that there are two scalar coefficients of internal energy of the contours in contour expansion operation, namely α and β. They determine continuity and smoothness of the contours [38]. Large values of the coefficients lead to less-expanded contours and an underestimation of detected boundaries. On the contrary, with lower values of the coefficients, the converging contours evolve strongly depending on the gradient field of the AFM height image. This leads to an overestimation of detected boundaries [37]. Therefore, during the process of foreground extraction, a proper value should be chosen to obtain optimized boundary detection of foreground features.

### Polynomial-fitting-based flattening

After mask area extraction in the foreground, polynomial fitting was applied to the background of AFM images. As mentioned earlier, if polynomial fitting was directly applied to the whole images without foreground feature exclusion, as was done previously [29,39–41], black/bright stripes will appear around the convex/concave features in the flattened images. To solve the problem, only the portions of scan lines in the background area were used for polynomial fitting.

**Figure 4:**
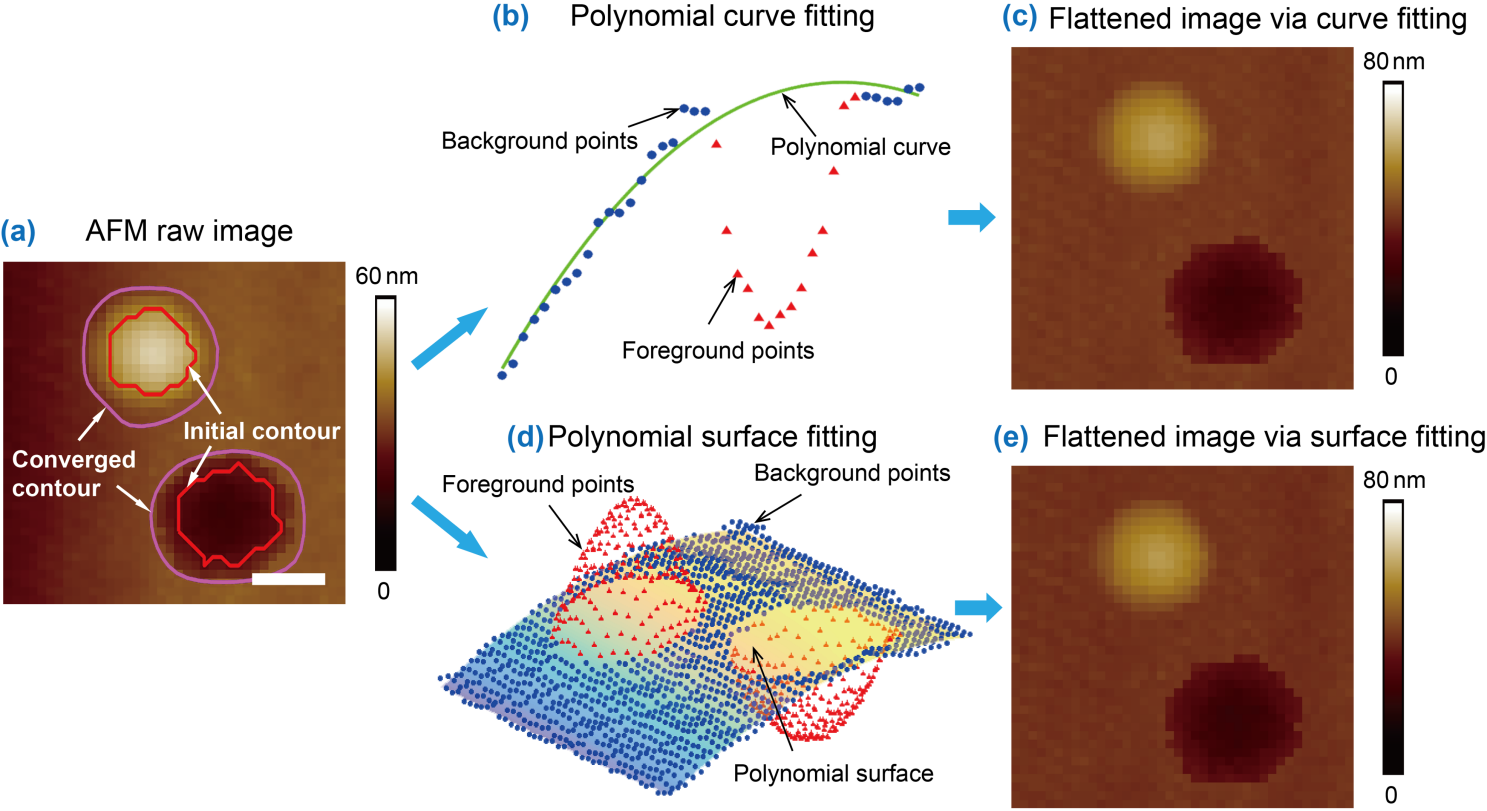
Demonstration of polynomial-fitting-based flattening for an AFM image. (a) A raw AFM image with segmented convex and concave features. (b) A third-order polynomial curve fitting of a scan line with the foreground portion excluded. (c) Flattened AFM image with the polynomial curve fitting based flattening. (d) Mesh plot of the raw image and the fitted third-order polynomial surface with the foreground features excluded. (e) Flattened AFM image with the polynomial surface fitting based flattening. Only the data points in the background were used for fitting. The scale bar is 100 nm.

Both polynomial curve fitting and surface fitting were applied, as demonstrated in Figure 4. For the raw AFM image shown in Figure 4a, the foreground features were first segmented and excluded prior to polynomial fitting. In the polynomial curve fitting based flattening, the portions corresponding to the background in each scan line were fitted as a third-order polynomial curve using the least square method, as demonstrated in Figure 4b. In the figure, the red solid triangular markers are data points in the foreground and are excluded in polynomial fitting. The blue solid circular markers are data points in the background area for polynomial fitting. The green line is the fitted polynomial curve, which was then subtracted from the scan line. By repeating the operation for each scan line, the AFM height image was flattened, as shown in Figure 4c. Similarly, in the polynomial surface fitting based flattening, the data points in background area were fitted as a third-order polynomial surface, as shown in Figure 4d, which was then subtracted from the raw image to get the flattened image (Figure 4e).

## Results and Discussion

In this section, a comparison of different flattening methods was first conducted. Sliding-window-based polynomial curve fitting (SWCF) and sliding-window-based polynomial surface fitting (SWSF) are presented, followed by discussion of how the sliding-window size and fitting direction influence the flattened images. Finally, image flattening was applied to the morphological characterization and image flattening.

### Comparison of different flattening approaches

**Figure 5:**
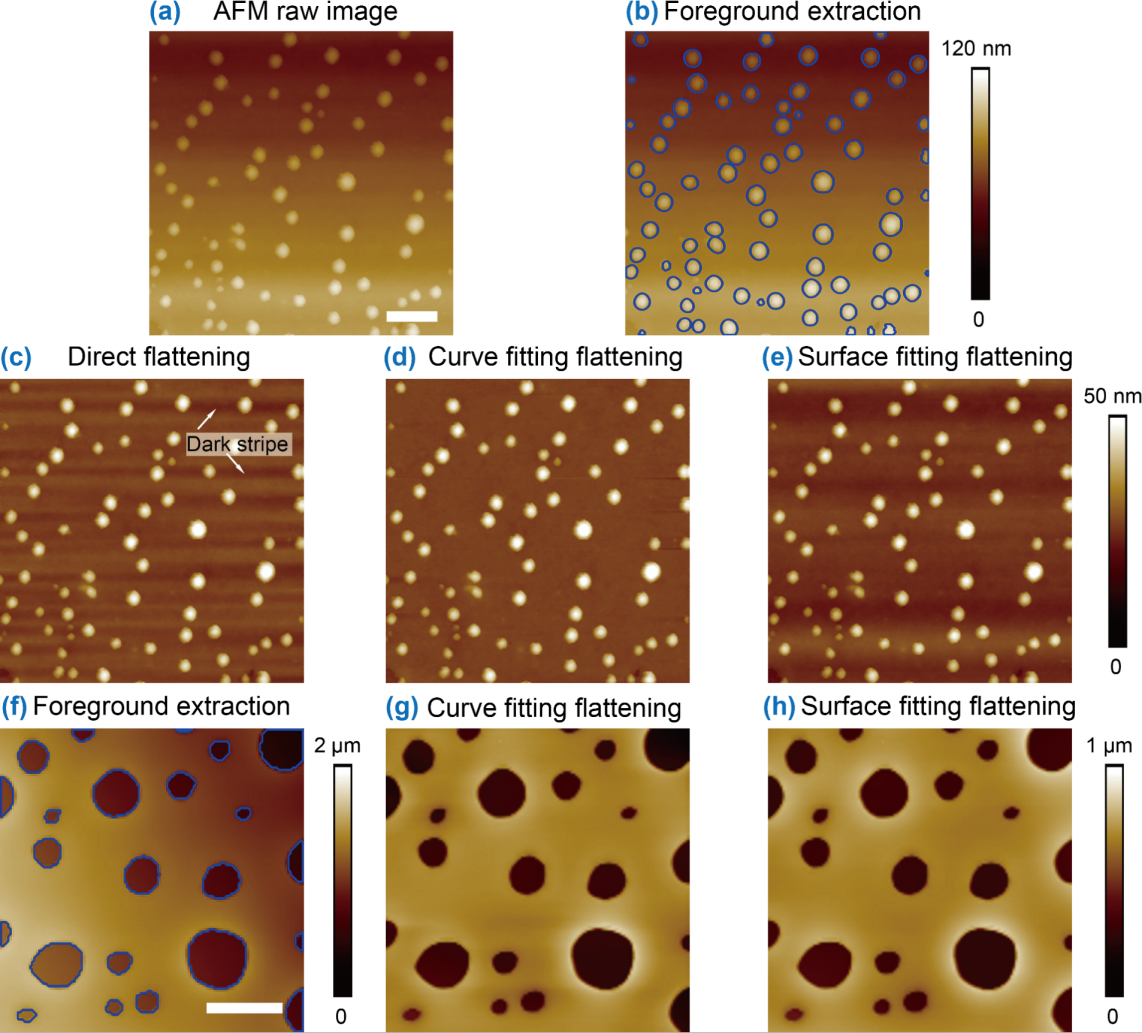
Comparison of the three different flattening approaches. (a) A raw nanobubble (NB) AFM image with tilting artifact. (b) Extraction of exclusion masks through contour expansion based image segmentation. (c) Flattened image using direct flattening without foreground mask area exclusion. This leads to dark stripes around the NBs. (d) and (e) are flattened images with mask-exclusion-based polynomial curve fitting and surface fitting, respectively. The former gives a desired flattening result. However, there is still severe fluctuation along the vertical direction due to the inadequate fitting in the latter. (f) A raw nanopit AFM image with detected exclusion masks. (g) and (h) are flattened images using mask-exclusion-based polynomial curve fitting and surface fitting, respectively. The results show that the proposed method can also flatten images with concave features. The scale bars in (a) and (f) are 500 nm and 10 µm, respectively.

For the NB AFM image shown in Figure 5a, the image segmentation was first implemented using the contour expansion method. The segmentation result is shown in Figure 5b. The detected foreground areas were excluded to get the background of the image. For the image, three different flattening approaches were applied. Figure 5c shows the results after directly applying the polynomial fitting to each scan line to the raw image without extraction of the foreground features. One can see that dark stripes [29] appear around the NBs.

The dark stripe artifacts can be avoided by employing the MEF approach. The third order polynomial curve fitting based MEF was first applied to the image. The flattened image is shown in Figure 5d. In the image, the dark stripes do not appear. However, in the flattened image obtained by the polynomial surface fitting based MEF (Figure 5e), the tilting artifacts were only partially corrected. The image still exhibits low frequency fluctuations. Practically, the background trend could be any arbitrary shape. A given order of a polynomial surface is inadequate to fit it.

Besides NBs, the proposed two-step scheme was also applied to AFM images with concave features. Figure 5f is an AFM height image of nanopits with detected boundaries (blue contours). After that, the polynomial curve fitting and polynomial surface fitting based MEF were applied to the image. The results are shown in Figure 5g and 5h. One can see that both methods can provide desired flattening for the image.

### Sliding-window-based polynomial curve fitting and surface fitting

**Figure 6:**
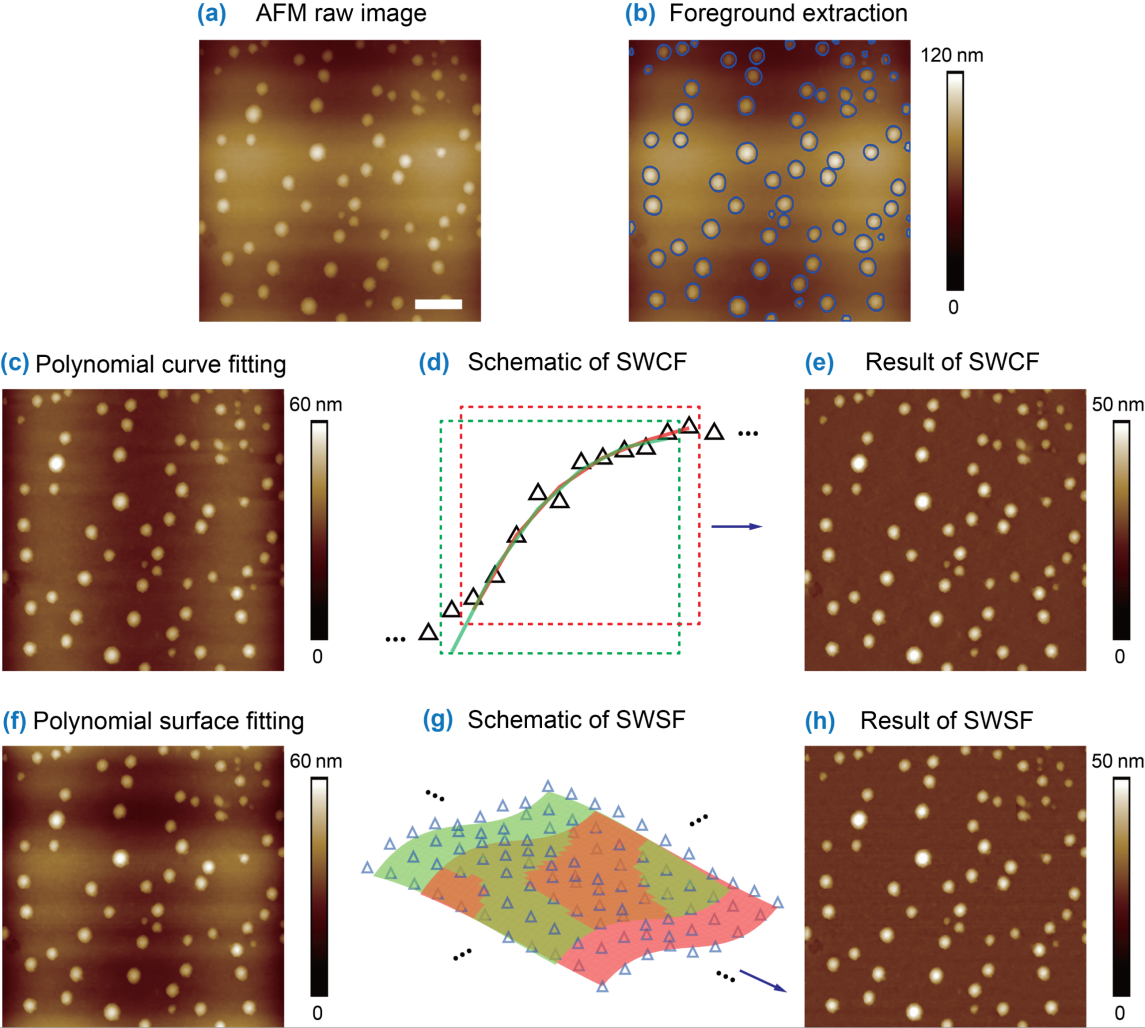
AFM image flattening with the sliding-window-based approaches. (a) A raw AFM height image with complex background along horizontal and vertical directions. (b) Extraction of exclusion masks through contour expansion operation of image segmentation. (c) and (f) are flattened AFM images by applying the polynomial curve fitting and polynomial surface fitting to the background area of the image, respectively. (d) and (g) Schematic of sliding-window-based curve flattening (SWCF) and sliding-window-based surface flattening (SWSF). (e) and (h) are flattened images obtained with the SWCF and SWSF, respectively. For the raw image, the direct application of MEF gives a poor flattening result, while SWCF and SWSF provide optimized results. The scale bar is 500 nm.

The order of polynomial fitting used for image flattening is directly related to the AFM background. Normally, third order or quadratic polynomial fitting are enough for AFM images with tilting or bowing types of artifacts. However, inadequate flattening occurs for AFM images with a complex background, as shown in Figure 6a. The corresponding segmentation result is shown in Figure 6b. The curve fitting based MEF was directly applied to the image. The result is shown in Figure 6c. It is clear that the direct application of polynomial curve fitting based MEF is inadequate. There are still some corrugate shaped artifacts in the flattened image. In this case, even increasing the fitting order of the polynomial curves could not guarantee the desired flattening result. Additionally, increasing the order of polynomial curves normally leads to Runge’s phenomenon [42–43].

In order to solve the problem of inadequate fitting, here we applied sliding-window-based [44] curve and surface flattening methods. In the sliding-window-based curve flattening (SWCF) method, for each scan line, a window with a certain width was first aligned to the starting point of a scan line. As demonstrated in Figure 6d, only the portion of the background points within the window was fitted as a third-order polynomial curve at each time of window sliding. After the polynomial fitting, the value on the polynomial curve at each point was recorded. The window was then slid along the scan line to the next point, followed by the corresponding polynomial fitting, until the end of the scan line. After that, the recorded value at each point was averaged and taken as the fitted value. By doing so, the fitted curve for each scan line can be determined and then extracted from the scan line. By applying the SWCF method, an optimized flattening result was obtained, as shown in Figure 6e.

The sliding-window operation can also be combined with polynomial surface fitting. The flattening result with the direct polynomial surface fitting is shown in Figure 6f. Obviously, the flattened image still displays heavy artifacts due to the inadequate fitting. Similar to the SWCF, the sliding-window-based surface flattening (SWSF) was applied to the image by iteratively sliding a two-dimensional window of a certain size along the horizontal and vertical directions, as demonstrated in Figure 6g. After that, a fitted surface was obtained and extracted from the raw image to obtain the flattened image (Figure 6h).

From this example, one can see that when AFM images exhibit a complex background trend, the direct application of MEF is not adequate. Either SWCF or SWSF should be applied. During the operation of sliding-window-based flattening, the sliding-window size and the fitting direction needs to be considered.

### Influence of sliding-window size

**Figure 7:**
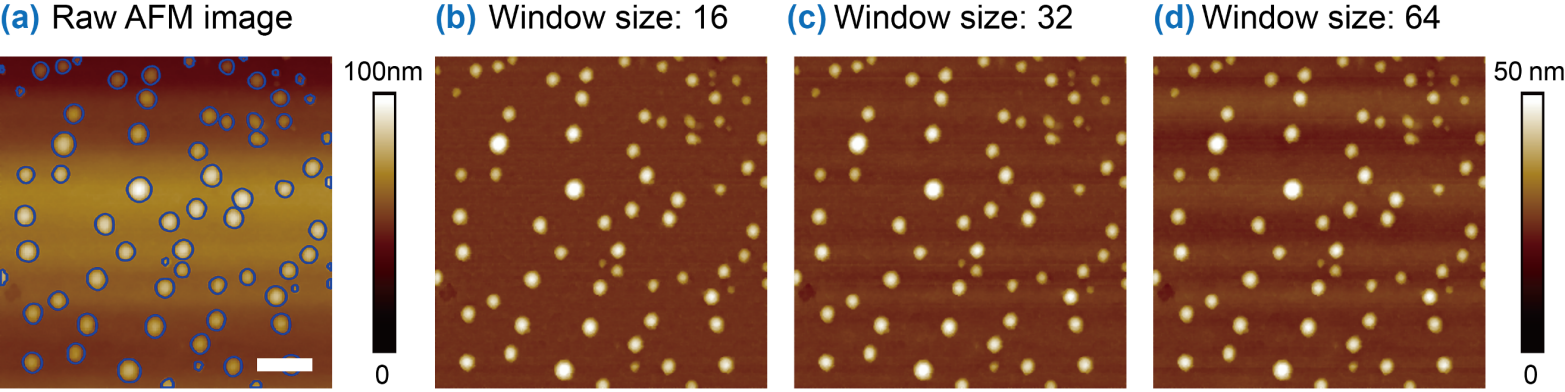
Comparison of image flattening with different sizes of sliding windows in the sliding-window-based surface flattening (SWSF) method. (a) A raw AFM image with foreground segmented. (b), (c), and (d) are flattened images with the sliding windows of 16 × 16, 32 × 32, and 64 × 64, respectively. With increasing window size, the corrugate-shaped artifacts appear. The scale bar is 500 nm.

In SWCF and SWSF, the size of the sliding windows is an important parameter, especially for SWSF. For the image shown in Figure 7a, SWSF was implemented with window sizes of 16 × 16 (Figure 7b), 32 × 32 (Figure 7c), and 64 × 64 (Figure 7d). It is clear that a smaller window size provides a better flattening result. With increasing window size, the corrugate-shaped artifacts gradually appear. That is because the larger windows include more data points, which in turn causes inadequate flattening. In the extreme case, the window has the exact the same size as the raw image. The flattening result will be exactly the same as shown in Figure 6f. In practice, the window size should be empirically determined according to the background complexity of the raw AFM images. For artifacts with relative lower frequency, larger window sizes are preferred. Otherwise, a smaller window size should be applied to obtain the optimized flattening result.

### Influence of fitting direction in sliding-window-based curve flattening

During scanning, AFM images are constructed line by line. The time required for each scan line is around 1 s along the fast scanning direction, which is much shorter than that of several minutes required for an entire image. As a result, the direction of drift is normally perpendicular to that of the fast scanning, namely, along the slow scan direction. Along individual scan lines, the drift is much lower. Here we investigate the influence of fitting direction on the performance of image flattening in SWCF.

**Figure 8:**
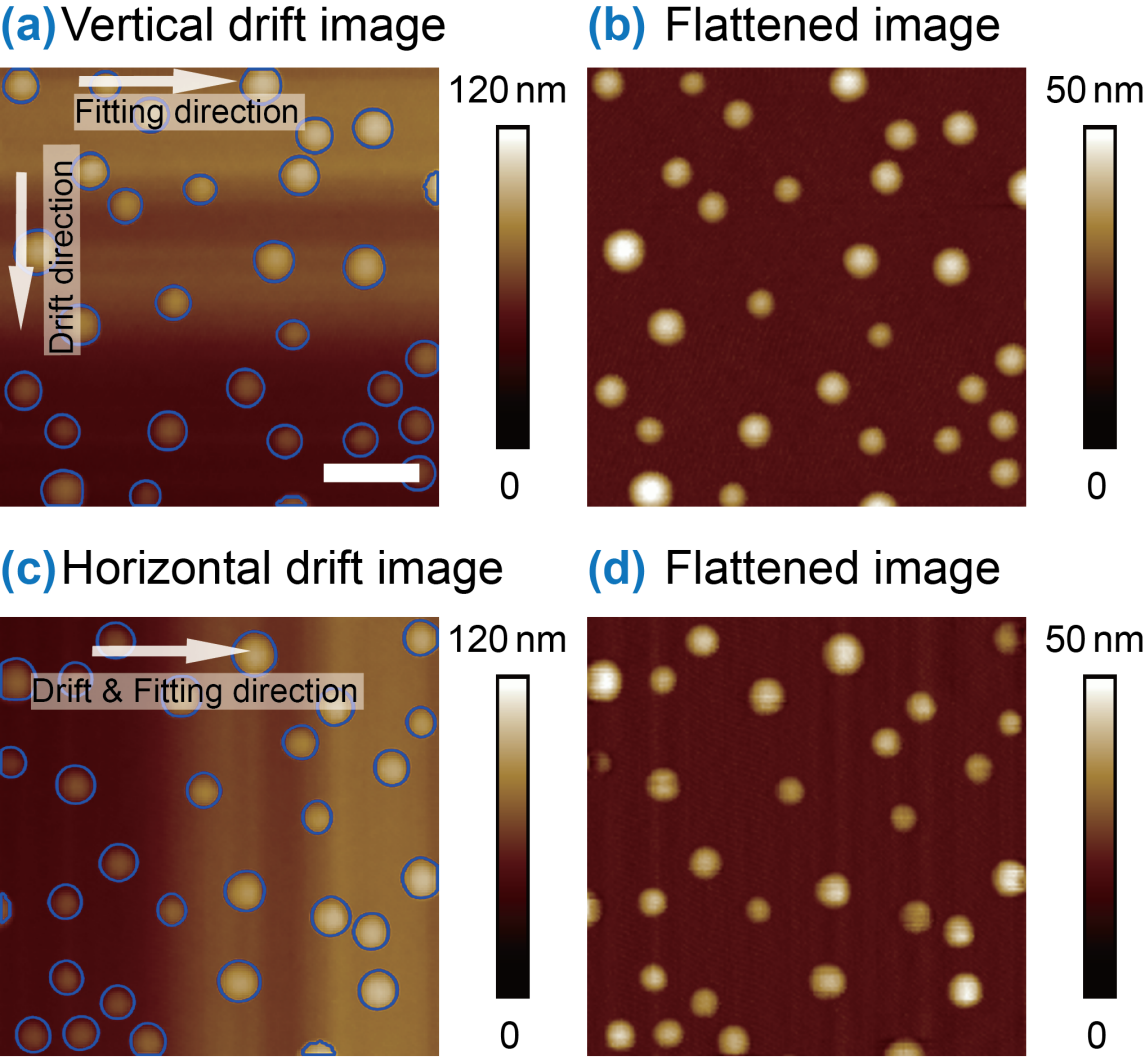
Influence of fitting direction on the flattened images. (a) and (c) are nanobubble images with vertical drift and horizontal drift, respectively. (b) and (d) are the corresponding flattened images with horizontal fitting direction. The optimized flattening result can be obtained when the drift direction and the fitting direction are perpendicular to each other.

Figure 8a shows a raw AFM image. The SWCF was then applied to individual scan lines along the horizontal direction (fast scanning direction). The flattened result is shown in Figure 8b. One can see that the SWCF along the horizontal direction gives an optimized image flattening. To test the influence of fitting direction relative to the drift direction, here the raw AFM image was rotated 90° clockwise (Figure 8c). The SWCF was then applied to the rotated image. In this case, the fitting direction is the same as that of the drift. The flattened image is shown in Figure 8d. Compared with Figure 8b, the flattened image still displays some corrugate-shaped artifacts. Through the example, one can conclude that to get an optimized flattening result, the fitting should be along the direction of lower drift, which is normally along the fast scanning direction.

### Morphological characterization with the flattened images

AFM images directly obtained from scanning contain artifacts. Therefore, image flattening needs to be implemented before analysis. Here we take an AFM image of a standard calibration grating as an example to validate the importance of image flattening in morphological characterization.

**Figure 9:**
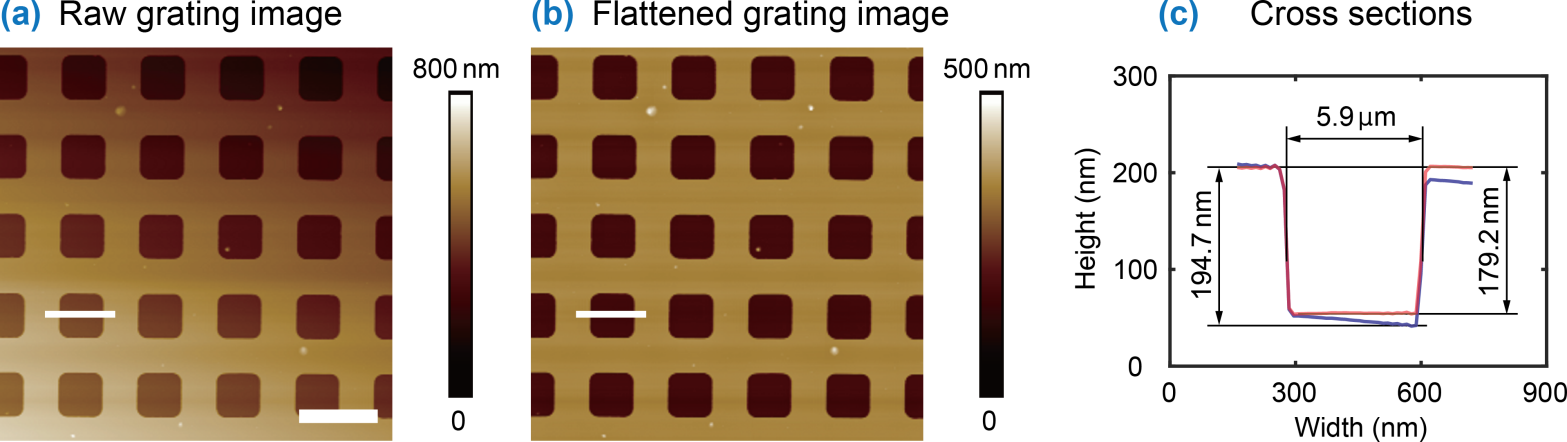
Morphological characterization of a standard AFM calibration grating with and without flattening. (a) The raw AFM image of the standard calibration grating. (b) Flattened image of the grating. (c) Comparison of cross sections of a micrometer-sized pit before and after the flattening. The blue curve shows the profile in raw image, while the red curve shows the profile in flattened image. The flattened image gives a better measurement of the depth of the pit. The scale bar is 10 µm.

The raw AFM image of the grating is shown in Figure 9a. The image exhibits a tilting artifact from bottom left to top right. By applying MEF, the image was flattened. The result is shown in Figure 9b, where the tilting was removed. A comparison of cross sections for a selected micropit from the raw image and the flattened image is shown in Figure 9c. Since the raw image contains tilting artifacts, the cross section (blue) is tilted. This gives an overestimation of the depth measurement, which is defined as the vertical distance between the highest and the lowest points across the pit. The measured depth from the raw image is 194.7 nm. For the flattened image, the tilting artifact is corrected and the base line of the cross section becomes flat. This gives a depth of 179.2 nm, which is closed to the quoted value of 180 nm. The relative error is 0.43%, which is much lower than that of 8.17% from the raw image.

### Image segmentation with the flattened AFM images

In addition to the morphological characterization, image flattening also facilitates segmentation of AFM images. AFM image segmentation is a process of extracting objects of interest in AFM images. It is a primary step for morphological analysis [3,38]. It is difficult to get a good segmentation result for AFM images with uneven background. The image flattening removes background artifacts and improves image segmentation.

**Figure 10:**
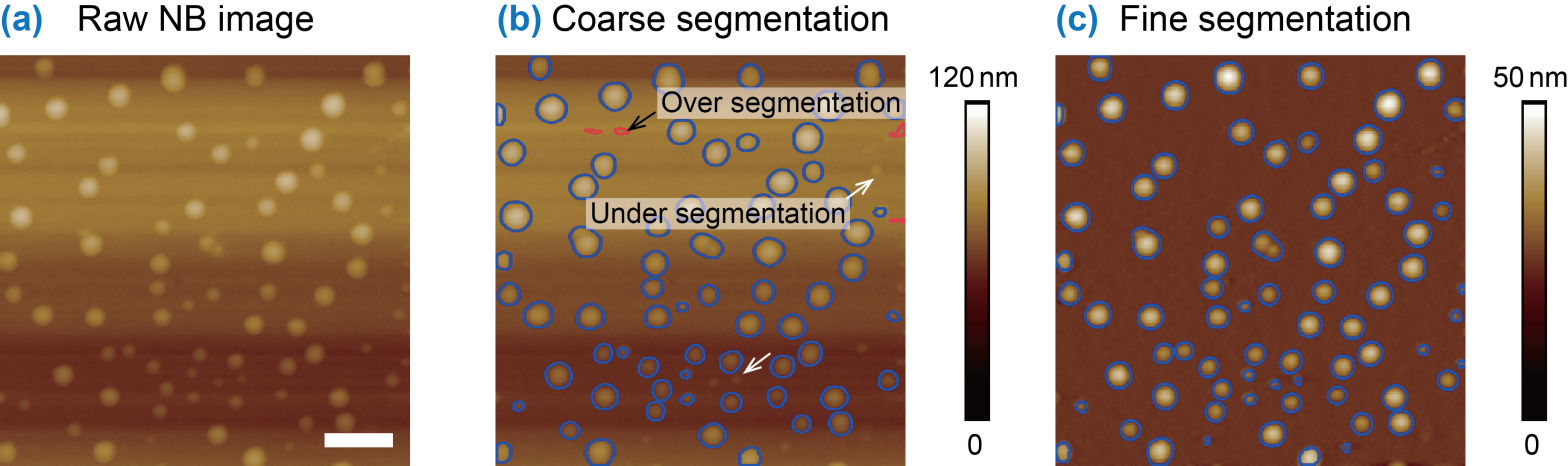
AFM image segmentation with flattened images. (a) A raw AFM image of nanobubbles (NBs) with artifacts in the background. (b) Segmentation of NBs with adaptive thresholding method, followed by the contour expansion operation on the raw AFM image. Both over-segmentation and under-segmentation occur. (c) Image segmentation on the flattened image. After image flattening, all NBs can be correctly segmented without any over-segmentation. The scale bar is 500 nm.

Here we take a NB image as an example. The raw AFM image shown in Figure 10a was first directly segmented with the contour expansion method. The result is shown in Figure 10b. Both over-segmentation and under-segmentation occur. For example, the areas enclosed by red contours belong to the background, as indicated by a black arrow. They are falsely detected as NBs, which is referred to as over-segmentation. Meanwhile, some tiny bubbles are not detected, resulting in the under-segmentation (indicated by white arrows in Figure 10b).

Here the proposed image flattening method was applied to obtain an optimized segmentation. First, the adaptive thresholding combined with contour expansion was applied to get a preliminary segmentation result (Figure 10b). The preliminarily segmented areas are taken as exclusion masks for MEF. In the flattened image, the segmentation was applied again. Since the image flattening removes background trend, the second image segmentation provides an improved result, as shown in Figure 10c. In the image, one can see that all NBs can be correctly segmented and over-segmentation is avoided.

## Conclusion

Flattening is a primary step for morphological analysis of AFM images. Here we developed a new scheme for optimized AFM image flattening in an automated manner. The scheme consists of two steps: automated extraction of exclusion masks in the foreground and polynomial fitting of the background in AFM images. In the mask area extraction, the adaptive thresholding and contour expansion operation were combined to achieve the automated segmentation of concave and convex features, which were then taken as exclusion masks. For images with tilting or bowing type of artifacts, polynomial fitting can be directly applied to the entire scan lines or the whole background areas with excluded foreground features. After polynomial fitting, the obtained polynomial curves or surfaces can then be subtracted from raw images to get flattened images. For images with a complex background trend, sliding-window-based polynomial curve and surface fitting were proposed and optimized flattening results were achieved. The influence of sliding-window size and direction of polynomial fitting on the flattened results were investigated. Finally, the role of image flattening was further validated in morphological characterization and image segmentation of AFM images. The results show that the proposed method can provide a more accurate measurement of feature dimensions and optimized image segmentation compared with the raw AFM images.
